# Undescribed Breast Blood Supply from the Brachial Artery: Circumflex Mammary Artery

**DOI:** 10.1007/s00270-025-04174-9

**Published:** 2025-09-09

**Authors:** Shinichi Hori, Atsushi Hori, Norifumi Kennoki, Tatsuya Nakamura, Ikuo Dejima, Akihiko Kumamoto, Tetsuro Sonomura

**Affiliations:** 1 Department of Interventional Radiology, Institute for Image Guided Therapy, Osaka, Japan; 2 The Department of Radiology, Wakayama Medical University, Wakayama, Japan

**Keywords:** Breast, Artery, Anatomy, Blood supply, Angiography, Carcinoma

## Abstract

**Purpose:**

Recent advancements in medical technologies have made trans-arterial treatment of breast cancer feasible. Consequently, understanding the vascular anatomies of breast cancers and axillary lymph node metastases has become indispensable for sophisticated treatments. The aim of this study was to determine the vascular anatomy of the breast, which is crucial for trans-arterial chemoembolization in patients with breast cancer.

**Materials and Methods:**

A total of 126 treatment-naive breast cancer patients were investigated to evaluate the arteries supplying breast tissues, tumours, and axillary lymph node metastases. The breast-supplying arteries were identified via angio-CT scans performed in the same examination room.

**Results:**

As described in anatomical textbooks, the main arteries supplying breast tissues and tumours are mammary branches arising from the internal thoracic artery and lateral thoracic artery. However, an undescribed serpiginous artery arising from the brachial artery distal to the subscapular artery and circumflex humeral artery was discovered in 46.0% of patients. This artery partially supplied breast tumours in 30.2% of the patients. The circumflex mammary artery is presumed to be a normal variant that is not pathologically enlarged due to tumour angiogenesis. Axillary lymph node metastases are supplied mainly by the lateral thoracic artery and thoracodorsal artery.

**Conclusion:**

We identified an undescribed serpiginous artery arising from the brachial artery distal to the subscapular artery or circumflex humeral artery, and we named this the circumflex mammary artery.

**Graphical Abstract:**

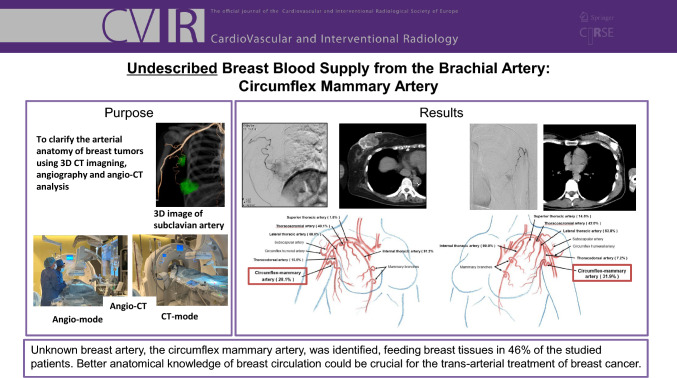

## Introduction

Trans-arterial treatment is initiated for patients with advanced breast cancer who are ineligible for standard therapies, including resection or chemotherapy [1, 2] [3]. On this basis, our group aimed to identify all anatomical variations in the arterial supply of breast tissue, as well as axillary lymph nodes, to ensure the adequate delivery of chemotherapeutic agents and embolic materials to target lesions. The major arterial supply to breast tissues comes from mammary branches arising from the internal thoracic artery and the lateral thoracic artery arising from the subclavian artery. However, the arteries supplying breast tissue have not been sufficiently examined in previous studies. Angio-CT images were analysed to determine the blood supply to breast tumours in 126 patients who were either unresectable or unable to receive chemoradiation treatment. In this study, we aimed to identify and report the incidence of arteries supplying breast cancer. A thorough understanding of these arteries is essential to achieve trans-arterial chemoembolization of breast cancer and axillary lymph node metastases.

## Materials and Methods

This study was approved by the institutional review board. Between January 2004 and June 2024, 126 consecutive patients with primary breast cancer who were unresectable or ineligible for chemoradiation therapy were enrolled. The arteries supplying the breast cancer and axillary lymph node metastases were retrospectively examined. A biopsy confirmed the presence of breast cancer in all patients. The mean age was 56 (range: 30–85) years. The tumour location was on the right side in 57 patients and on the left side in 69 patients and was distributed as follows: superior medial quadrant (A area) in 24.6% (31/126), inferior medial quadrant (B area) in 6.3% (8/126), superior lateral quadrant (C area) in 48.4% (61/126), and inferior lateral quadrant (D area) in 20.6% (26/126). The mean tumour size was 49 mm (range: 8–177 mm). Eighty patients had axillary lymph node metastases (41 on the right side and 39 on the left side), with a mean size of 20 mm (range: 10–67 mm).

For the selective insertion of a microcatheter into mammary branches, 3D-CT vascular images were reconstructed via the volume rendering technique of the subclavian artery and its branches prior to angiography.

The subclavian artery and its branches were identified via digital subtraction angiography (DSA) following a standard femoral artery approach via a 4Fr catheter. Under the guidance of DSA images and 3D-CT images, a preshaped 2.0 Fr microcatheter (Estream IGT, Toray Medical, Tokyo) was advanced to the branches of the subclavian artery. During selective arterial contrast injection into each branch following the DSA study, a CT scan was taken to confirm the blood supply to the breast tissues, tumours, and axillary lymph node metastases. Two hybrid angio-CT systems were used for this study (Toshiba, Infinix, Tokyo, Japan, and Canon Alfinix, Tokyo, Japan). For selective arterial CT, iomeprol (300 mg/ml) was injected at a rate of 0.05 to 0.5 ml/sec and in a volume ranging from 0.25 to 5.0 ml via a contrast injector dedicated to a microcatheter (MICA30, Clinical Support, Izumisano, Japan). A selective infusion of chemotherapy drugs was followed by embolization with a superabsorbent polymer microsphere.

## Results

**Fig. 1:**
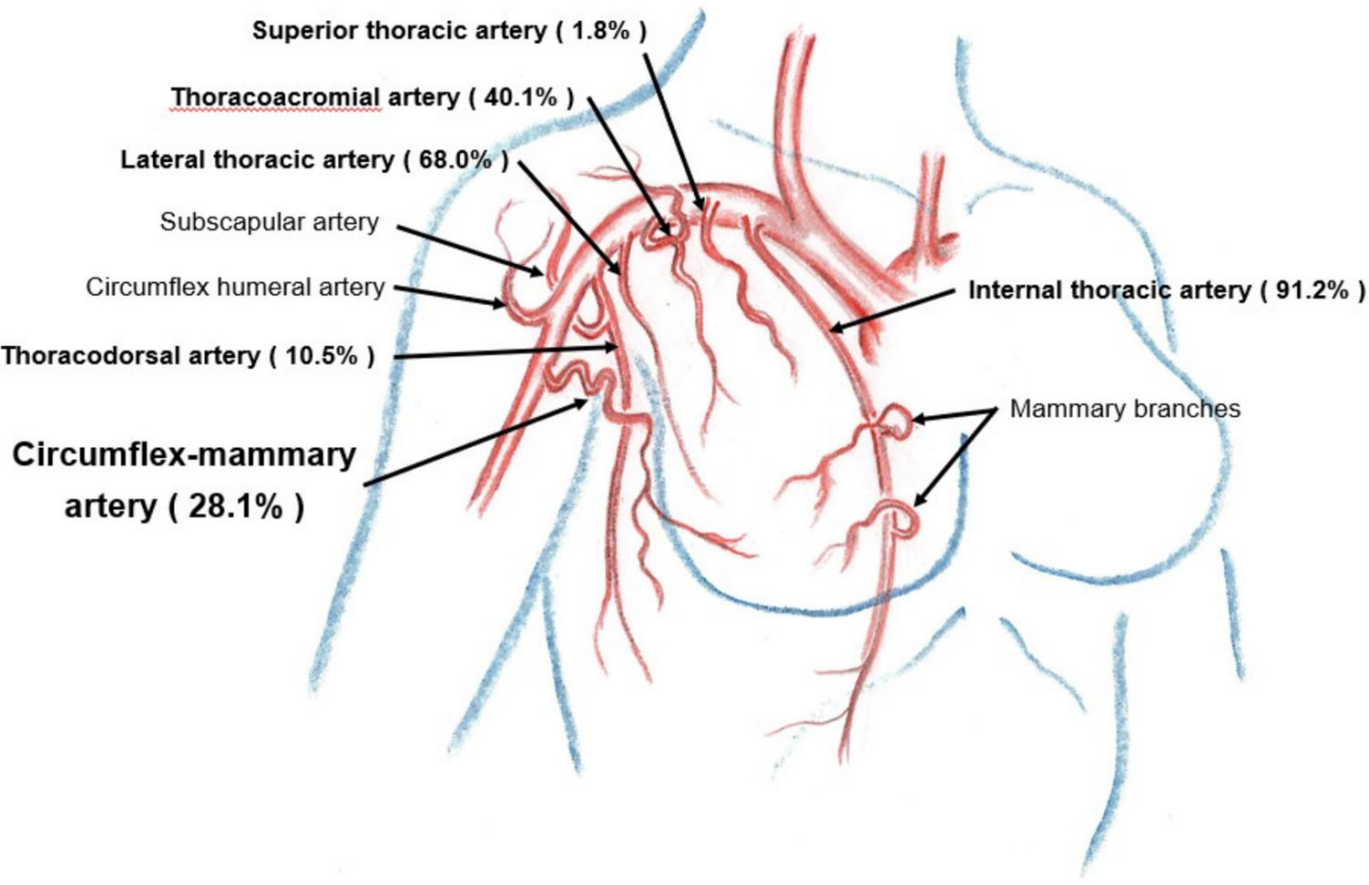
Breast tumour-supplying arteries on the right side

**Fig. 2:**
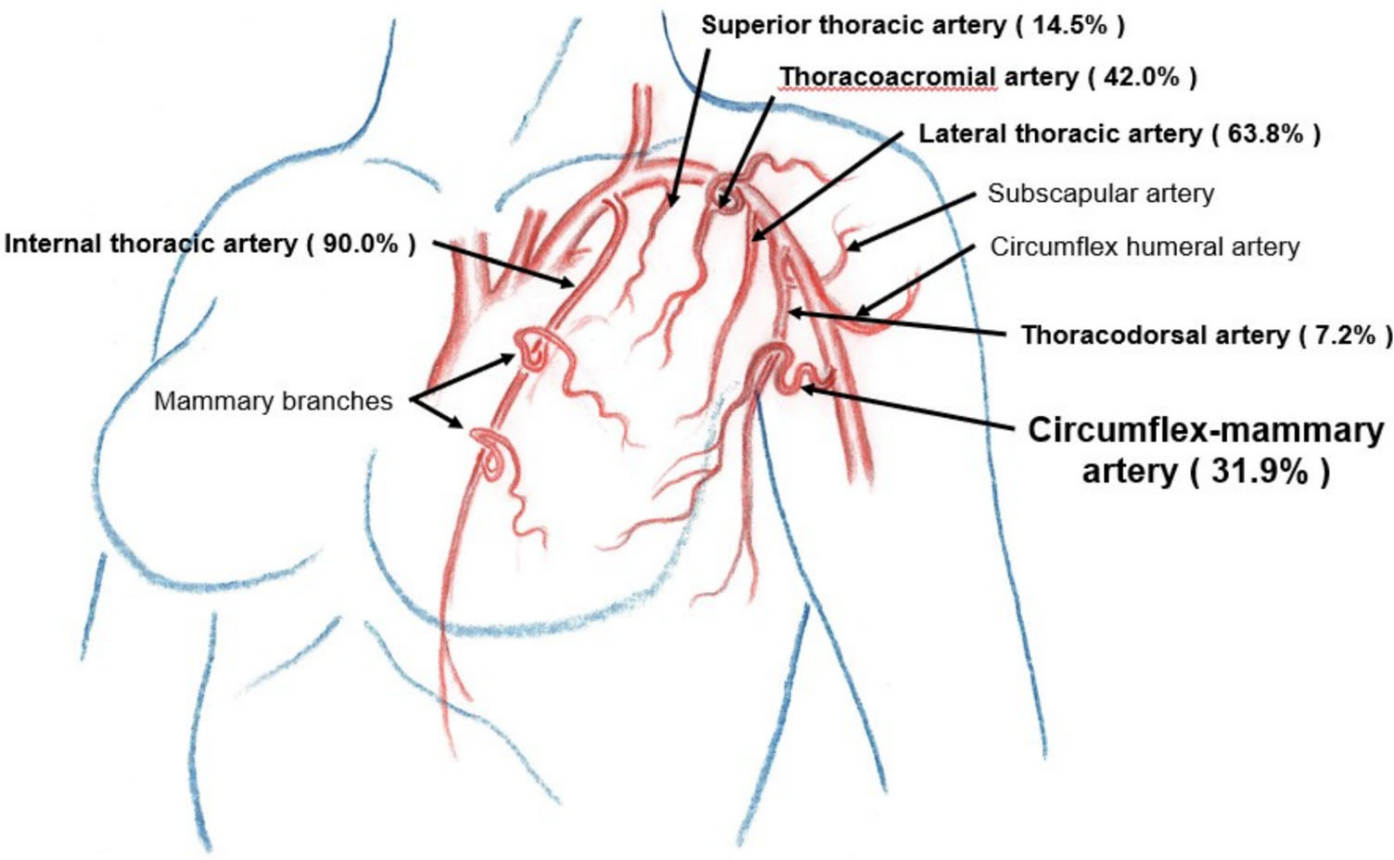
Breast tumour-supplying arteries on the left side

**Fig. 3:**
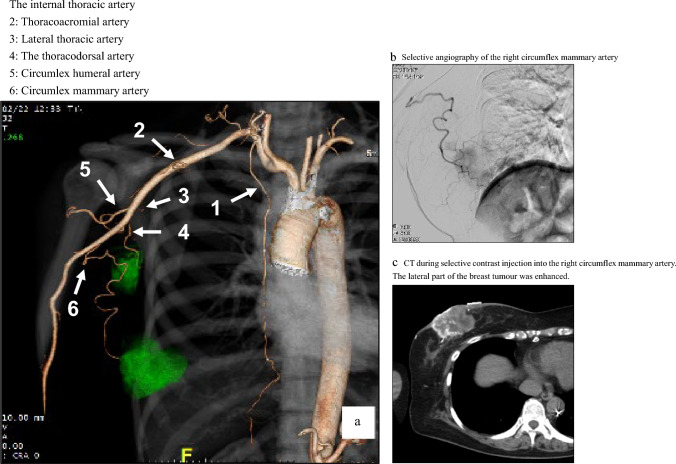
Right breast angiography and selective arterial CT. Arrows The internal thoracic artery 2: Thoracoacromial artery 3: Lateral thoracic artery 4: The thoracodorsal artery 5: Circumlex humeral artery 6: Circumlex mammary artery a. Angio-image of the right subclavian artery via the volume rendering technique, b. Selective angiography of the right circumflex mammary artery. c. CT during selective contrast injection into the right circumflex mammary artery. The lateral part of the breast tumour was enhanced

**Fig. 4:**
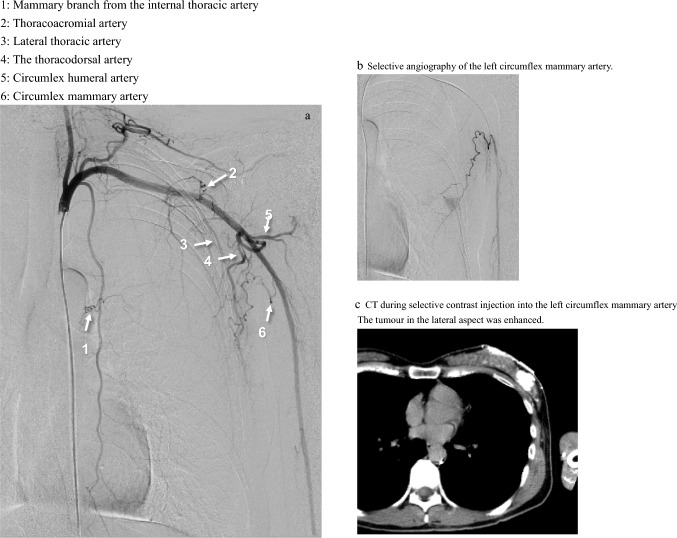
Left breast angiography and selective arterial CT. **a** Left subclavian digital subtraction angiography, Arrows 1: Mammary branch from the internal thoracic artery 2: Thoracoacromial artery 3: Lateral thoracic artery 4: The thoracodorsal artery 5: Circumflex humeral artery 6: Circumflex mammary artery **b** Selective angiography of the left circumflex mammary artery, **c** CT during selective contrast injection into the left circumflex mammary artery. The tumour in the lateral aspect was enhanced

As described in anatomical textbooks, the arteries supplying breast tissues are mammary branches arising from the internal thoracic artery, superior thoracic artery, thoracoacromial artery, lateral thoracic artery, and thoracodorsal artery. A previously undescribed artery was identified arising from the brachial artery distal to the subscapular artery and circumflex humeral artery in 46.0% (58/126) of the patients (right side in 49.1% (28/57) and left side in 43.5% (30/69)). The blood supply to breast tissues through this artery was confirmed in all 58 patients by selective angio-CT in this study. We propose the name *circumflex mammary artery* for this vessel (Figs. 1, 2, 3, and 4).

The right-sided breast arteries that supply the breast tumours are illustrated in Fig. 1 and 3. Axillary lymph node metastases detected on diagnostic CT as enlarged nodes were all enhanced by selective contrast infusion on angio-CT. The arteries supplying right axillary lymph node metastases were the lateral thoracic artery (68.3%, 28/41), thoracodorsal artery (60.1%, 25/41), thoracoacromial artery (22.0%, 9/41), and circumflex mammary artery (17.1%, 7/41).

Similar results were obtained for the left breast tumour-supplying arteries, as shown in Figs. 2 and 4. The arteries supplying left axillary lymph node metastases were the lateral thoracic artery (76.9%, 30/39), thoracodorsal artery (64.1%, 25/39), circumflex mammary artery (35.9%, 14/39), thoracoacromial artery (5.1%, 2/39), and internal thoracic artery (2.6%, 1/39).

On both sides, the arteries supplying the breast tumours were the internal thoracic artery (90.5%, 114/126), lateral thoracic artery (65.9%, 83/126), thoracoacromial artery (41.3%, 52/126), circumflex mammary artery (30.2%, 38/126), superior thoracic artery (8.7%, 11/126), and thoracodorsal artery (8.7%, 11/126). For axillary lymph node metastases, the supplying arteries were the lateral thoracic artery (72.5%, 58/80), thoracodorsal artery (62.5%, 50/80), circumflex mammary artery (26.3%, 21/80), thoracoacromial artery (13.8%, 11/80), and internal thoracic artery (1.3%, 1/80).

## Discussion

Regional chemotherapy is an attractive concept that aims to deliver higher doses of drugs to the targeted tumour [2, 4, 5]. Nevertheless, trans-arterial treatment of breast cancer has not been recognized as a standard treatment. Recent progress in imaging modalities and catheter techniques may allow for more sophisticated locoregional treatments for breast cancer [1–3]. Understanding the arterial supply to breast tissues and tumours is crucial for achieving technical success and good clinical outcomes.

The mammary branches from the internal thoracic artery penetrate the chest wall near the sternum and pass through the subcutaneous tissue to the medial aspect of the breast. The lateral aspect is variably supplied by branches from the subclavian artery, including the lateral thoracic artery [4, 6, 7]. Our study focused on the blood supply to patients with breast cancer and reported similar results. The thoracoacromial artery should also be considered a contributor to the breast’s vascular supply. The pectoral branch of the thoracoacromial artery descends between the major and minor pectoral muscles, and in some cases, penetrates the major pectoral muscle to supply the breast tissue.

As described in previous reports [4, 6, 8], there are many anatomical variations in arteries that supply breast tumours. In Huelke's anatomical investigation [9], there was no description of blood supply from the brachial artery. Even in a recent anatomical study of the axillary artery performed via CT angiography, the circulation from the brachial artery to the breast tissue was not described [9]. In Doughty's clinical investigation using selective dye infusion techniques, they noted the possibility of perfusion from a further branch of the subclavian or axillary artery [6].

An analysis of the arteriograms and dye infusion performed by McCarter revealed extreme individual variation in the arteries supplying the lateral aspect of the breast [4]. In a study of selective intra-arterial chemotherapy, the arterial supply for breast cancer was investigated by estimation of CT images during selective intra-arterial contrast injection [10]. They did not identify the arterial supply from the brachial artery but noted that not all tumours were enhanced through the internal thoracic and lateral thoracic arteries.

The presence of this newly identified artery, the circumflex mammary artery, explains the unclear descriptions in previous reports; it arises distal to the subscapular trunk and extends to the breast tissues beyond the axilla, forming serpiginous shapes. The incidence was found to be 46% of all cases. The ratio of blood supply from the identified artery to breast tumours was 30.2%.

We presume that the circumflex mammary artery represents a normal anatomical variant rather than a pathologically enlarged vessel resulting from tumour-associated demand or angiogenesis. In this study, some circumflex mammary arteries did not supply breast tumours; however, imaging analysis in healthy patients is necessary for further studies.

Recently, hybrid CT/angiography (angio-CT) systems have been developed for accurate intra-arterial drug administration [11]. Using this system, we simultaneously obtained selective angiography images and selective arterial infusion images of breast tumours. This system allows estimation of the territory of individual arteries to the chest wall and breast tissues, making accurate trans-arterial treatment of breast cancer possible. Thus, it is extremely important to recognize the unknown circulation to achieve good results from trans-arterial treatment of breast cancer. Although additional radiation exposure compared with conventional angiography must be considered, arterial infusion CT is well worth the effective administration of drugs to the target lesion, especially in patients in critical condition due to advanced breast cancer.

Breasts develop from the thickening of the ectoderm called the mammary ridge during the fifth week of gestation. The mammary ridge, called the milk line, extends from the axilla to the groyne. The persistence of the ridge at any location along the milk line may result in accessory breast tissue [12]. The incidence of accessory breast tissue is 0.4%-0.6% in women [13]. This ectopic breast tissue is commonly present in the axilla [12, 14]. For this reason, the presence of the circumflex mammary artery could be due to remnant breast tissue in the axilla.

The blood supply to axillary lymph node metastases was also demonstrated in this study. The lateral thoracic and thoracodorsal arteries are major vessels involved in lymph node metastasis, but the circumflex mammary artery or thoracoacromial artery sometimes supply metastatic nodes. Trans-arterial chemoembolization of axillary lymph node metastasis can be performed simultaneously with primary breast tumours.

## Conclusion

A previously unknown breast artery, the circumflex mammary artery, was identified, suppling breast tissues to 46% of the studied patients. A better understanding of the anatomy of the breast circulation could be crucial for the trans-arterial treatment of breast cancer.
